# Altered protein homeostasis in cardiovascular diseases contributes to Alzheimer’s-like neuropathology

**DOI:** 10.1007/s00395-025-01109-w

**Published:** 2025-05-07

**Authors:** Nirjal Mainali, Meenakshisundaram Balasubramaniam, Sonu Pahal, W. Sue T. Griffin, Robert J. Shmookler Reis, Srinivas Ayyadevara

**Affiliations:** 1 Bioinformatics Program, University of Arkansas for Medical Sciences and University of Arkansas at Little Rock, Little Rock, AR 72205 USA; 2 Central Arkansas Veterans Healthcare Service, Little Rock, AR 72205 USA; 3 Department of Geriatrics, Reynolds Institute on Aging, University of Arkansas for Medical Sciences, Little Rock, AR 72205 USA

**Keywords:** Cardiovascular disease, Alzheimer’s disease, Protein aggregates, Crosslinking studies, Leave-one-out analysis

## Abstract

Cardiovascular diseases (CVDs) are the leading cause of death worldwide. CVD is known to increase the risk of subsequent neurodegeneration but the mechanism(s) and proteins involved have yet to be elucidated. We previously showed that myocardial infarction (MI), induced in mice and compared to sham-MI mice, leads to increases in protein aggregation, endoplasmic reticulum (ER) stress in both heart and brain, and changes in proteostatic pathways. In this study, we further investigate the molecular mechanisms altered by induced MI in mice, which were also implicated by proteomics of postmortem human hippocampal aggregates from Alzheimer’s disease (AD) and cardiovascular disease (CVD) patients, vs. age-matched controls (AMC). We utilized intra-aggregate crosslinking to identify protein–protein contacts or proximities, and thus to reconstruct aggregate “contactomes” (nonfunctional interactomes). We used leave-one-out analysis (LOOA) to determine the contribution of each protein to overall aggregate cohesion, and gene ontology meta-analyses of constituent proteins to define critical organelles, processes, and pathways that distinguish AD and/or CVD from AMC aggregates. We identified influential proteins in both AD and CVD aggregates, many of which are associated with pathways or processes previously implicated in neurodegeneration such as mitochondrial, oxidative, and endoplasmic-reticulum stress; protein aggregation and proteostasis; the ubiquitin proteasome system and autophagy; axonal transport; and synapses.

**Supplementary Information:**

The online version contains supplementary material available at 10.1007/s00395-025-01109-w.

## Introduction

Neurodegenerative diseases such as Alzheimer’s disease (AD), Parkinson’s disease (PD), and Huntington’s disease (HD) are characterized by the accumulation of misfolded proteins within the neurons and glial cells of brain parenchyma [82]. Misfolding leads to proteotoxic aggregation of proteins, a key characteristic of aging which has also been implicated in age-associated conditions including hypertension, myocardial ischemia, and cardiovascular disease [4]. The cellular pathways responsible for clearance of misfolded proteins and their aggregates, necessary to maintain or reestablish protein homeostasis, are also disrupted during neurodegenerative diseases, cardiovascular diseases, aging, and hypertension [2, 4]. The two main aggregate-abatement pathways are the ubiquitin–proteasome system (UPS) and autophagy; consequences of their disruption include oxidative stress, endoplasmic reticulum (ER) stress, and mitochondrial stress in the affected organ [58].

Cardiovascular diseases (CVDs) are the leading cause of mortality globally [61] and include conditions such as atherosclerosis, coronary artery disease (CAD), and arterial hypertension (AH). Atherosclerosis involves the thickening and hardening of arterial walls, which impact the cardiovascular system and other organs [44]. AH often lacks conspicuous symptoms but is a key risk factor for myocardial infarction (MI), stroke, renal failure, and peripheral vascular diseases [45]. CAD is the narrowing or blockage of coronary arteries due to fatty plaque deposits, which hamper the delivery of blood, oxygen, and nutrients to the heart muscle, potentially leading to MI and death [38]. While CVD encompasses a broad range of heart and vessel conditions, MI is a specific, acute event caused by blocked blood flow to the heart [72]. MI diagnosis is relatively urgent, focusing on acute symptoms and biomarkers, whereas CVD diagnosis involves assessing long-term risk factors and chronic conditions [72]. Mouse models of MI are valuable in cardiovascular research as they help elucidate CVD mechanisms and the physiological effects of MI, replicating key clinical features of the condition in humans [66, 72].

The processes leading to protein aggregation are not well defined, but it is widely accepted that aggregates are initiated by oligomerization of seed proteins, which in AD are primarily Aβ_1-42_ and hyperphosphorylated tau (hP-tau) fragments [79] but may also include hyperphosphorylation of glial fibrillary acidic protein (GFAP) [27], α-synuclein [90], transactive response DNA-binding protein 43 (TDP-43) [89], and triggering receptor expressed on myeloid cells 2 (TREM2) [43]. Tau fragments and Aβ_1-42_ oligomers coalesce with a variety of other proteins chiefly through hydrophobic interactions, and recruit other misfolded or unfolded proteins to form large, dense aggregates that evade clearance by protein homeostasis machinery [42]. Intrinsically disordered proteins (IDPs) are preferentially aggregated in AD brain [3], since they require no further impetus to misfold. In the serum proteomes of AD patients, Aβ-specific aggregates comprise ~ 75% IDPs, while tau-specific aggregates consist of 35% IDPs [3].

We have established novel methods to model the interior architecture of aggregates, by chemical crosslinking with small, aggregate-permeating “click chemistry” molecules that facilitate recovery and identification of linked peptide pairs [5]. Knowledge of protein–protein contacts or proximities enables interactome modeling of the aggregate interior, first conducted with human SY5Y-APP_Sw_ neuroblastoma cells [5] and subsequently with aggregates isolated from AD and age-matched-control (AMC) hippocampi [6]. We used conventional machine-learning algorithms, neural networks trained on empirical data, and a novel leave-one-out-analysis (LOOA) procedure to guide graph modeling and ranking of proteins and their interfaces with respect to predicted influence on aggregate formation, connectivity, and stability [5, 6, 63]. It is of particular interest that many proteins other than Aβ_1-42_ and hP-tau contribute early in the formation and cohesion of aggregates, and thus offer promising targets for disruption of pathogenic aggregation in the initial phases of AD and other neurodegenerative diseases.

MI impedes oxygen supply to the brain, thus inducing hypoxia comparable to the effects of high altitude, chronic obstructive pulmonary disease, asthma, or cerebrovascular disease [87]. In animal and clinical studies, the molecular mechanisms implicated in post-hypoxia cognitive impairment include glycolysis, oxidative stress, calcium overload, inflammation, mitochondrial injury, apoptosis, and pathogenic effects of Aβ and tau [8, 40, 56, 106]. Aβ and tau accumulate in the brain and can be detected in serum or plasma as well as cerebrospinal fluid [25, 36, 98]. Mitochondria are among the first organelles affected by hypoxia, as they require oxygen for ATP formation. When the oxygen level falls, ATP also decreases, leading to disruptions in protein transport, synthesis, and folding; at the same time, generation of reactive oxygen species (ROS) increases [39]. Prolonged and severe hypoxia has also been linked to loss of dopaminergic neurons, hyperphosphorylation of α-synuclein and tau, and consequent memory impairment [56, 106].

In the present study, we analyzed proteomics of mouse and human hippocampal aggregates and used crosslinking data from human hippocampal aggregates to construct nonfunctional interactomes that summarize average structures of intra-aggregate protein assemblies. These “consensus interactomes” enabled us to define aggregate constituents that implicate organelles, processes, and functional pathways which may mediate the increased risk of cognitive decline after MI or cardiovascular disease. Both myocardial and cerebral aggregates include many proteins previously found to be enriched in AD brain aggregates [64]. We propose that CVD/MI may promote protein misfolding and aggregation of protein constituents resembling those that drive AD, predisposing to brain dysfunction and cognitive impairment. We compared hippocampi from cardiovascular disease (CVD) patients to those from age-matched controls. We also contrasted a transgenic-mouse model of AD amyloidopathy (BRI-Aβ_42_ mice) to wild-type mice and compared experimental-MI mice to sham-MI mice.

## Materials and methods

### Isolation of aggregates and analysis of their proteins

Frozen tissues were minced and homogenized with mortar and pestle at 0 °C in lysis buffer: 20-mM HEPES buffer pH 7.4, 0.3-M NaCl, 2-mM MgCl_2_, and 1% (v/v) NP40, containing inhibitors of proteases and phosphatases [CalBiochem]. Homogenates, after sonication on ice (3 × 10 s), were centrifuged 5 min at 2000 × g to remove debris. Lysate protein concentrations were determined (Bradford Assay, Bio-Rad). After 15-min centrifugation at 14,000 × g, pellets containing aggregates were resuspended in 0.1-M HEPES buffer, 1% sarcosyl (v/v) and 5-mM EDTA, and centrifuged 30 min at 100,000 × g. Pellets and supernatants (detergent-insoluble and -soluble fractions, respectively) were resuspended in Laemmli buffer at 95 °C and electrophoresed on polyacrylamide gels; 1-mm slices were incubated in trypsin for LC–MS/MS analysis as described [2].

### Isolation of Aβ-specific sarcosyl-insoluble aggregates from AD, CVD, and AMC human brain tissues

Briefly, flash-frozen tissue from AD, CVD or AMC caudal hippocampus (*N* = 3 per group) was pulverized in a mortar and pestle pre-cooled on dry ice. After 5-min low-speed centrifugation (2200 × g), supernatant protein was quantified with Bradford reagent (Bio-Rad) and equal protein portions were analyzed. To isolate Aβ-specific aggregates, the samples were incubated with DYNAL Protein-G magnetic beads coated with monoclonal antibody raised against a synthetic Aβ_1–17_ peptide (ab11132; Abcam). Bound aggregates were rinsed 3 times, eluted, and brought to 1% (v/v) sarcosyl, 0.1-M HEPES, and 5-mM EDTA, to which was added a cocktail of protease and phosphatase inhibitors (Sigma Aldrich PPC1010). Sarcosyl-insoluble aggregates were pelleted by ultracentrifugation (90 min at 100,000 × g), resuspended in 20-mM phosphate-buffered saline (pH 7.5), and processed for cross-linking.

### Chemical cross-linking of insoluble aggregates

A modified cross-linking reagent, propargyl amine, was prepared as a stock solution in DMSO and added to insoluble aggregate fractions to achieve a final concentration of 5 mM and incubated 30 min at 22 °C. Cross-linking reactions were quenched with 50-mM Tris–HCl (pH 8.0), and the samples were centrifuged 90 min at 100,000 × g at 4 °C to remove unbound cross-linker. Cross-linked aggregates were then incubated in 8-M urea and 122-mM dithiothreitol buffer for 30 min at 37 °C. To this reaction mix, 40-mM iodoacetamide was added and incubated 20 min at 22 °C in the dark. To the reduced cross-linked sample, 10 units of trypsin (Pierce) was added, and ammonium bicarbonate to 150 mM; after 14 h incubation at 37 °C, digestion was quenched by addition of acetic acid to 3% (v/v). Trypsin-digested cross-linked peptides were then desalted on a 1-cc C18 column (Sep-Pak, Waters) containing 50 mg resin, and evaporated to dryness (Speed-Vac, ThermoFisher). The samples were then reconstituted in a tenfold molar excess of biotin crosslinker/azide solution, 0.25-mM TBTA, 250-mM CuSO_4_, and 5-mM Tris-phosphine (TCEP) buffer. After 2-h incubation at 4 °C, biotinylated cross-linked peptide pairs were captured on streptavidin-coated magnetic beads as described [5]. Bound cross-linked peptides were eluted in buffer containing 50% (w/v) acetonitrile and 0.4% (v/v) trifluoro-acetic acid, which were then identified by LC–MS/MS [5].

### Graph modeling of protein–protein interaction (PPI) networks in the Aβ-specific aggregate interactome

Cross-linked peptide pairs were identified and their relative abundances estimated, with a modified version of Xlink-Identifier software [5]. A list of proteins previously identified in Aβ-specific aggregates was used as a reference database, to calculate *m*/*z* values for all possible tryptic-peptide pairs that could arise by cross-linking proteins within aggregates. To reduce the complexity of cross-linking data, only peptide-peptide pairs attaining ≥ 5 spectral hits per sample were included for further analysis. To reduce false positives, only protein–protein pairs identified in all three individual brain samples per group were carried forward for further data processing. All data analyses were performed using Linux scripts developed in-house. The results from Xlink-Identifier were first processed by GePhi Windows-based software to calculate network descriptors, including the number of directly interacting partners (degree) of each protein.

### Meta-analysis of functional annotation clustering with DAVID

DAVID (http://david.abcc.ncifcrf.gov) analyzes the lists of differentially expressed genes or differentially abundant proteins by seeking enrichment of functional-annotation terms (also called “gene ontology” or GO terms) associated with each entry in the list, beyond that which would be expected for a random list of proteins from the same species. Functional-annotation clustering eliminates much of the redundancy that arises in GO analyses, in large measure by combining multiple resources for gene or protein annotation. Terms that are associated with the same, or largely overlapping, sets of gene/protein names are presumed to refer to the same biological properties and are, therefore, clustered together. The outputs are selectable, but include (as in Tables 2 and 3) a name assigned each cluster to represent its biological meaning, *N* (the number of genes associated with any annotation cluster), the fold-change (factor by which the term or cluster is enriched), and the Benjamini-adjusted *P* value of annotation enrichment (using the Benjamini–Hochberg estimate of the false-discovery rate) to correct for analysis of multiple correlated terms.

### *siRNA knockdowns and thioflavin-T staining in SY5Y-APP*_*Sw*_* cells*

To knock down the expression of target genes, human neuroblastoma cell line SH-SY5Y-APP_Sw_, expressing a human APP transgene containing the ‘‘Swedish’’ familial-AD mutations, was maintained as previously described [5]. Briefly, the cells were grown to 70% confluence, trypsinized, and sub-cultured in 96-well plates (at 30,000–40,000 cells/well), supplemented with antibiotic-free DMEM containing 10% (v/v) FBS (Atlanta Biologicals GA). Hypoxia was conducted as previously described [74]; after 7 h of hypoxia at 37 °C, Lipofectamine 3000 (Invitrogen) was used to transfect cells with a short interfering RNA (siRNA; Sigma Aldrich), targeting each candidate gene of interest, viz., *DCTN1* (SASI_Hs01_00065675), *KIF5C* (SASI_Hs01_00337806), PSMD2 (SASI_Hs01_ 00042153), *RAB1A* (SASI_Hs01_00060095), *RAC1* (SASI_Hs01_00015565), *UBB* (SASI_Hs01_ 00201423), and *VDAC1* (SASI_Hs01_00012464). Transfected cells, including control cells transfected with a random-sequence siRNA construct (Sigma Aldrich), were maintained 48 h at 37 °C prior to thioflavin-T staining. To assess amyloid-like aggregates, siRNA-treated cells were incubated with 0.1% (w/v) thioflavin T in phosphate-buffered saline and imaged in a Keyence automated-stage fluorescence microscope. The total aggregate fluorescence per cell was calculated using the Fiji plug-in to ImageJ.

### Statistical analyses

The inter-group comparisons were assessed for significant differences in means by standard Fisher *t* tests with full Bonferroni correction for multiple endpoints, requiring an adjusted *P* value (alpha) < 0.05. In a few comparisons with low *N* (< 6), a heteroscedastic *t* test was used to allow for unequal or inadequately determined variances. These significance tests are thus quite stringent, and at least as conservative as 1-way ANOVA. Significance of annotation-term enrichment was calculated using the Benjamini–Hochberg procedure to estimate and limit the false-positive rate. Full Bonferroni correction fails to allow for correlations among outcomes and thus is excessively conservative when addressing complex datasets. The comparisons of ratios were assessed for significance using Chi-squared or Fisher Exact tests, the choice of which depends on the number of events/observations per cell.

## Results

### Cerebral aggregates caused by Aβ accumulation and myocardial infarction share many of the same proteins.

Proteomic analyses of cerebral aggregates from 12-month-old C57BL/6N mice overexpressing Aβ_1-42_ (BRI-Aβ_42_) [69], relative to age-matched wildtype mice (each *N* = 3), identified 783 proteins that are enriched at least twofold (BRI-Aβ_42_/WT ratio > 2) and 793 depleted (ratio < 0.5) in BRI-Aβ_42_ total aggregates. We performed similar proteomic analyses of cerebral aggregates isolated 7 days after left-coronary-artery (LCA) ligation to mimic myocardial infarction (MI) in C57BL/6 J mice *vs.* sham-MI mice, all at the same average age (4.5 months). After MI, 277 aggregate proteins were enriched (ratio > 2) while 439 proteins were depleted (ratio < 0.5) relative to sham-MI mice (Supplementary Fig. 1).

**Fig. 1:**
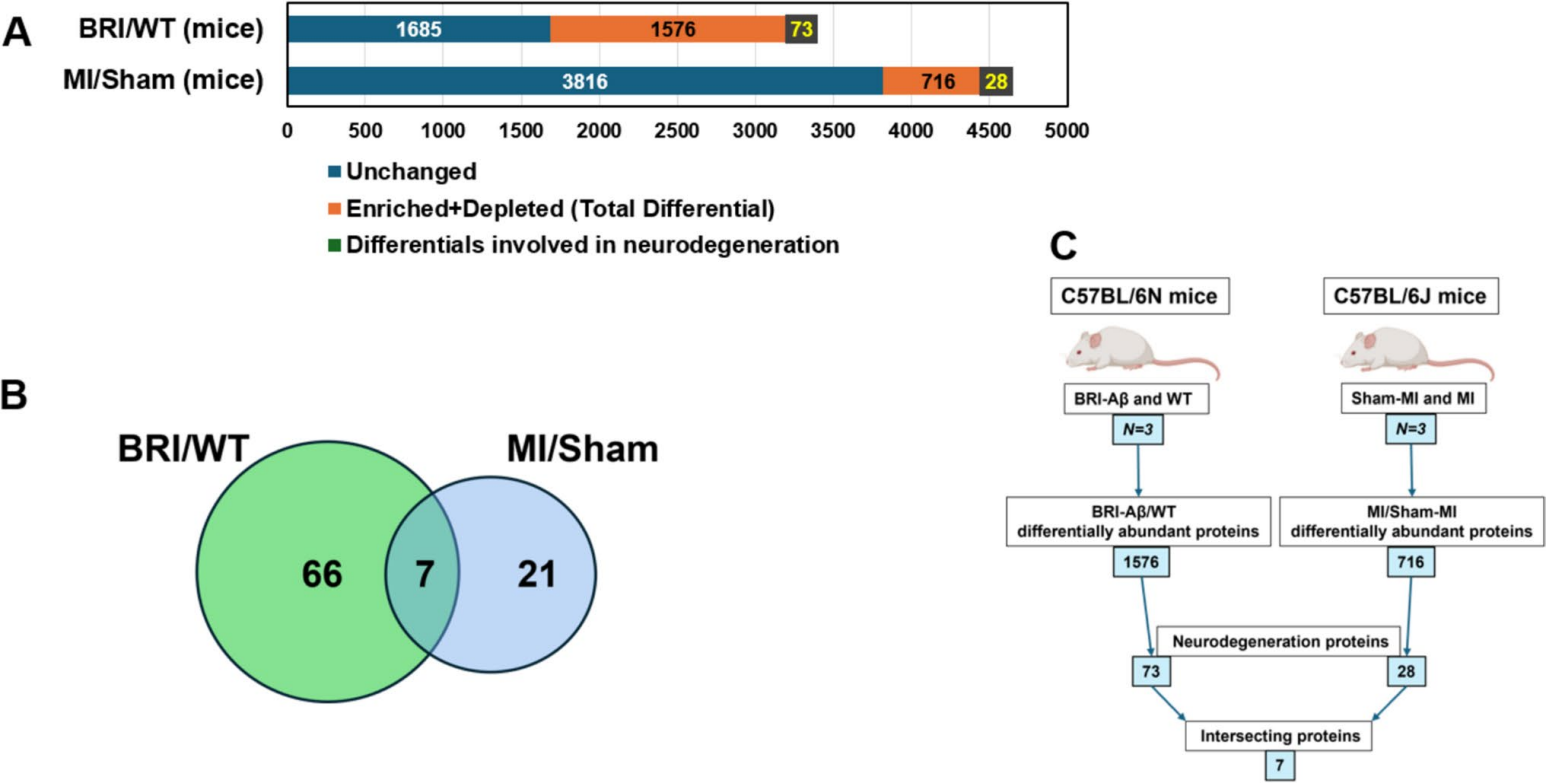
Proteomic and GO analyses for proteins differentially abundant in MI *vs*. sham-MI aggregates, and in BRI-Aβ_42_ *vs*. wildtype C57BL/6N mice. Aggregates were isolated from hippocampi of MI, sham-MI, BRI-Aβ_42_, and wildtype (WT) mice and their proteomes analyzed. Based on these comparisons (MI *vs.* sham-MI, and BRI-Aβ_42_ *vs.* WT), we identified differentially abundant proteins (enriched + depleted) in BR-Aβ_42_ and MI aggregates compared to their respective controls. **A** 1685 proteins were unchanged, 1576 proteins were differential, and 73 of these differential proteins were annotated as involved in neurodegeneration in BRI-Aβ_42_ mice relative to WT. Similarly, 3816 proteins were unchanged, 716 proteins were differential, and 28 differential proteins were involved in neurodegeneration in MI mice relative to sham-MI mice. For BRI-Aβ_42_ *vs.* WT, *χ*^2^ (2) = [494], *P* < 2.2E – 16; whereas for MI *vs.* sham-MI, *χ*^2^ (2) = [5149], *P* < 2.2E – 16 employing Chi-squared tests. **B** Venn diagram showing 7 differentially abundant shared by both comparisons. **C** Flowchart of mouse animal-model comparisons and results

The 1576 aggregate proteins that were differentially abundant in BRI-Aβ_42_ mice relative to non-transgenic controls were submitted for analysis of functional-annotation-term enrichment using DAVID [18]. Terms with enrichment scores > 4 are considered highly significant [113]; these included ATP-binding, microtubule binding, pleckstrin homology (conferring the potential to bind PIP_3_), ubiquitin-like protein conjugation, cytoskeleton, RNA binding, kinases, pathways of neurodegeneration, etc., each with Benjamini *P* < 1.7 × 10^**‒**5^*.* This included 73 (4.6%) proteins associated with neurodegeneration, including AD, PD, HD, amyotrophic lateral sclerosis (ALS), and prion disease (Fig. 1A and Supplementary Fig. 1).

Similarly, functional-annotation-term enrichment analysis of 716 proteins differentially abundant in mouse MI *vs*. sham-MI cerebral aggregates implicated mitochondrial roles, ATP binding, calcium signaling, and ribosomes as highly enriched descriptors, each with Benjamini *P* < 1 × 10^**‒**7^*.* In this comparison, 28 proteins (3.9%) were tagged with “neurodegeneration pathways” for a relatively modest enrichment score of 1.32 (Fig. 1A and Supplementary Fig. 1). Seven proteins annotated for “neurodegeneration pathways” were shared between the sets differentially abundant in BRI-Aꞵ_42_ mice and in MI-induced brain aggregates (Fig. 1B).

### Hippocampal aggregates isolated by Aβ_1–42_ immuno-pulldown (IP), from AD and CVD patients vs. AMC, show differentially abundant neurodegeneration-pathway proteins shared by AD and CVD

The proteins from AMC, AD, and CVD human hippocampi (each *N* = 4) were extracted (description of tissues, Supplementary Table 1), and IP conducted with antibody to Aβ. Proteomic analyses of these Aβ-specific aggregates compared hippocampi from AMC, AD, and CVD individuals. Relative to AMC, CVD aggregates were enriched for 1335 proteins and depleted for 110 proteins. Similarly, AD aggregates were enriched for 583 proteins and depleted for 259 proteins, in AD relative to their AMC controls (Supplementary Fig. 2).

**Fig. 2:**
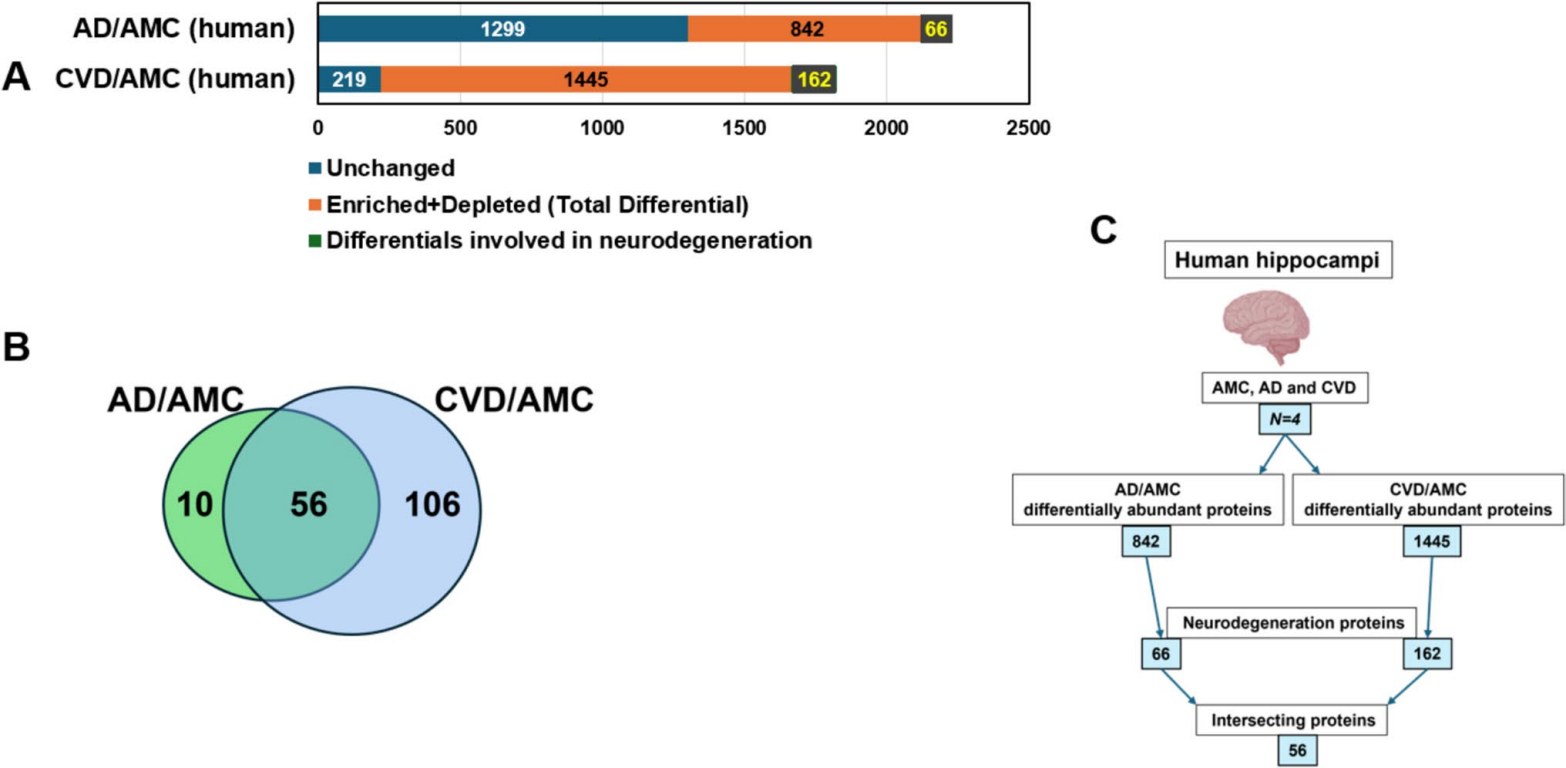
Proteomic and GO analyses for human hippocampal-aggregate proteins differentially abundant in AD and CVD, each relative to age-matched controls (AMC). Aggregates were isolated from hippocampal tissues of AD, CVD, and AMC individuals, and subjected to proteomic analyses. Comparisons of AD vs. AMC and CVD vs. AMC aggregate proteins identified those enriched or depleted in AD and CVD, relative to AMC. **A** Comparing AD to AMC aggregates, 1299 proteins were unchanged, 842 proteins were differentially abundant, and 66 differential proteins (7.8%) were annotated as involved in neurodegeneration. Comparing CVD to AMC aggregates, 219 proteins were unchanged, 1445 proteins were differential, and 162 differential proteins (11.2%) were annotated as involved in neurodegeneration. For AD vs. AMC, *χ*^2^ (2) = [794], *P* < 2.2E – 16; for CVD *vs.* AMC, *χ*^2^ (2) = [1657], *P* < 2.2E – 16 by Chi-squared tests. **B** Venn diagram showing 56 common proteins from differentially abundant AD/AMC and CVD/AMC comparisons. **C** Flow chart for AMC, AD, and CVD human hippocampal-aggregate comparisons and results

Functional-annotation-term enrichment analysis of the 842 aggregate proteins differentially abundant in AD relative to AMC, implicates terms including ribosome; actin, GTP, and ATP-binding; intermediate filament; neurodegeneration pathways; transport, folding and localization of proteins; microtubules; ubiquitination; and mitochondria, each with Benjamini *P* < 1 × 10 ^– 5^*.* This list contained 66 (7.8%) aggregate proteins involved in neurodegeneration (Fig. 2A and Supplementary Fig. 2).

Similarly, 1445 proteins differentially abundant in CVD relative to AMC aggregates imply enrichment for involvement of ATP, GTP, and actin binding; pathways of neurodegeneration; protein folding; chaperones; mitochondria; glycolysis; Kreb’s cycle; oxidative phosphorylation; ubiquitination; diabetic cardiomyopathy; macro-autophagy, etc., each with Benjamini *P* < 1 × 10^**‒**6^ Of the proteins in this list, 162 (11.2%) are involved in neurodegeneration pathways (Fig. 2A and Supplementary Fig. 2)*.*

Remarkably, 56 of the 66 neurodegeneration-annotated proteins that were differential in AD vs. AMC aggregates (85%), were also included among the 162 neurodegeneration-related proteins differential for CVD vs. AMC, of which they comprise ~ 35% (Fig. 2B).

### Proteins with more interacting partners, in Aβ-specific aggregate interactomes from CVD and AD relative to AMC, are involved in neurodegeneration-related functions

**Fig. 3:**
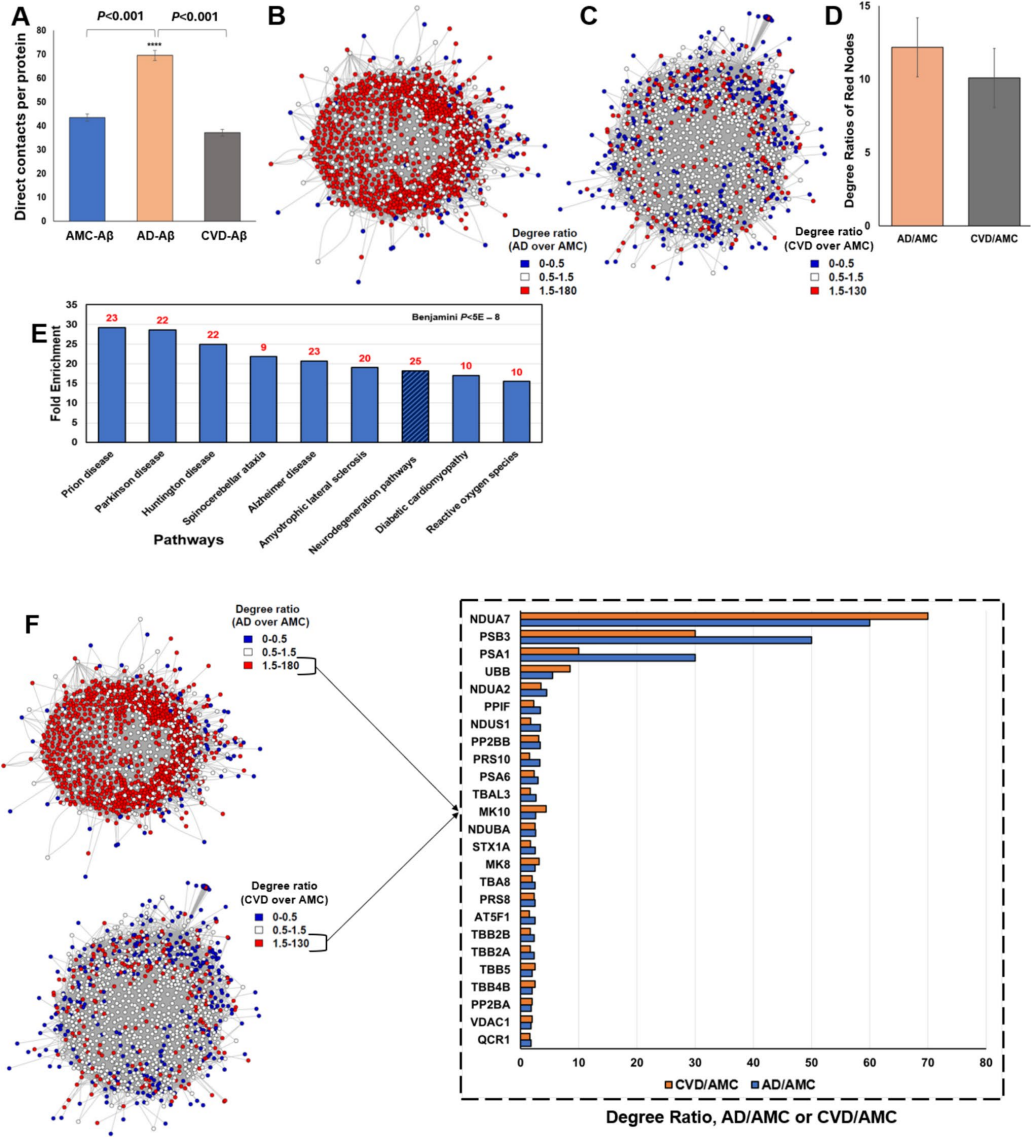
Aβ-IP aggregate interactome analysis from AMC, AD, and CVD hippocampi. Protein foci were recovered after immuno-pulldown (IP) from AD, CVD, and AMC hippocampal tissue using Aβ antibody and crosslinking with chemical crosslinkers; sarcosyl-insoluble aggregates were then isolated from them. After LC–MS/MS analysis, we used an R program to visualize the contactomes and determine the degree (number of direct interactions) of each protein. Degree ratios were determined for AD vs. AMC and CVD vs. AMC. **A** Mean degree (direct contacts) of all interactome proteins: 42 for AMC, 70 for AD, and 38 for CVD. AD interactions per protein (mean ± SEM) differed from AMC or CVD at *P* < 0.001 by 2-tailed heteroscedastic *t* tests. **B** Visualization of aggregate interactome shared by AD and AMC; red nodes have AD/AMC degree ratios ≥ 1.5, and blue nodes have ratios < 0.5 (less abundant in AD). **C** Aggregate interactome shared by CVD and AMC is displayed as in **B**, with node colors here indicating CVD/AMC degree ratios. **D** Mean ± SEM degree ratios were calculated for proteins with more interacting partners (degree) than AMC, in CVD (grey) or AD (orange). **E** KEGG pathway analysis, using 182 common proteins from those most differential in AD/AMC and CVD/AMC comparisons, implicate 25 proteins involved in neurodegeneration (Alzheimer’s, Parkinson’s, Huntington’s, Prion disease, etc*.*) with Benjamini-adjusted *P* value < 0.01 for annotation enrichment in DAVID (https://david.ncifcrf.gov/home.jsp). Numbers over bars indicate the number of differential aggregate proteins in each category. **F** Degree ratios are plotted for comparisons of shared differential proteins contrasting AD/AMC (blue bars) or CVD/AMC (orange bars)

We calculated the number of interacting partners of proteins in the AMC, AD, and CVD aggregate interactomes. While AMC and CVD had similar average numbers of interactions per protein (no significant difference), AD had ~ 65% more interactions than AMC (*P* < 0.001; Fig. 3A)**.**

We then analyzed Aβ-IP aggregate interactomes from AMC, AD and CVD hippocampi, and ranked differential proteins by the ratio of interacting-partner counts (i.e., the degree ratio), contrasting AD vs. AMC and CVD vs. AMC. We visualized the AD/AMC and CVD/AMC interactomes using R code developed in-house. Blue hubs in Fig. 3B, C, represent proteins that have at least twofold lower degree in either AD or CVD relative to AMC (degree ratio < 0.5), while red hubs indicate proteins with higher degree (degree ratio > 1.5) than AMC; white hubs indicate proteins with similar degree ratios in AD or CVD relative to AMC (0.5 < degree ratio < 1.5). Most AD-aggregate proteins had ≥ 1.5 times the degree of AMC constituents, as reflected by the very large number of red nodes (Fig. 3B), while CVD/AMC had lower degree ratios as indicated by far fewer red nodes despite a similar interactome complexity (Fig. 3C). This is consistent with an overall lower connectivity of the CVD interactome, featuring fewer indirect (e.g., secondary) interactions than AD. However, considering only red nodes in the AD and CVD interactomes, we calculated that the average degree ratio of red nodes (ratios > 1.5) is similar in CVD/AMC and AD/AMC (Fig. 3D).

There were 182 differential proteins with degree ratios of at least 1.5 in both AD/AMC and CVD/AMC interactome comparisons. Functional-annotation-term enrichment for these proteins reveals that out of 58 clusters, only the neurodegeneration descriptor had a significant enrichment score (> 4) of 7.5, with Benjamini *P* < 3 × 10^**‒**6^. Of these 182 total differential proteins, 25 (14%) were annotated as neurodegeneration-related; nearly all of those (23–24 of 25) are shared by AD, PD, HD, and prion disease; and 20 are also shared by ALS. Twelve proteins are tagged as related to reactive oxygen species (ROS), while 9 are shared by spinocerebellar ataxia (Fig. 3E). Figure 3F shows the top 25 differential proteins common to AD/AMC and CVD/AMC comparisons, with their respective degree ratios. This indicates that, despite different cell and tissue locations, aggregates specific to AD or CVD collect essentially the same complement of neurodegeneration-related protein families or pathways.

### Proteins involved in important cellular functions are sequestered in the aggregates of cardiovascular disease and Alzheimer’s disease brains

We combined the 56 proteins shared between AD and CVD individuals’ Aβ-specific aggregates (Fig. 2D) with seven proteins shared between BRI and MI mouse aggregates (Fig. 1D), for proteomic meta-analyses. To this list we added 25 differential proteins from the Aβ-specific AD/AMC and CVD/AMC interactome-degree comparisons; after removal of 12 duplicates, our list comprised 76 highly differential and influential proteins. We conducted KEGG pathway analysis [49] within DAVID (https://david.ncifcrf.gov) to visualize results in the context of neurodegeneration pathways (code: hsa05022). Many differentially abundant proteins (deep green boxes) are components of cellular pathways previously implicated in neurodegeneration. Significant enrichment was observed for accumulation of aggregates, ubiquitin-proteasomal system (UPS), glutamatergic synapse, ER stress, mitochondrial dysfunction, oxidative stress, autophagy, Wnt signaling, AGE-RAGE signaling pathway, and axonal transport (Supplementary Fig. 3A–J). These proteins are also compiled in Table 1 along with their functions.

**Table 1:** Detailed summary of 76 differentially influential proteins in AD vs. AMC and CVD vs. AMC comparisons

Annotation	Proteins	Functions
Mitochondria	AT5F1, ATPB, ATPG, ATPO	ATP production
	NDUA2, NDUA7, NDUBA, NDUS1, NDUAS2, NDUAS7, NDUV2, HCD2	Electron transport and proton pumping
	QCR1, QCR2	Oxidative phosphorylation
	COX2/MT-CO2	Cytochrome c oxidase subunit
	VDAC1, VDAC2, VDAC3	Outer mitochondrial membrane proteins
Endoplasmic reticulum	PP2BA, PP2BB	Calcium dependent protein phosphatases
Calcium signaling	KCC2B, KCC2D, KCC2G, KPCA, KPCB, KPCG	Calcium dependent kinases
	SERCA2/ATP2A2	Calcium transporter from cytosol to ER
	RYR3	Calcium release from ER
Synapse	GRIA2	Glutamate receptor
	KIF5C	Synaptic transmission
	SYUA	Synaptic vesicle trafficking
	DLG4, RAC1	Synaptic plasticity
	STX1A	Synaptic fusion
	HTRA2	Caspase activity
Apoptosis	MK1, MK3, MK8, MK10, KC1A	Regulation of neuronal apoptosis
Proteostasis	PRS10, PRS8, PSMD2	26S proteasome
	PSA1, PSA6, PSB2, PSB3	20S proteasome
	UBA1, UBB, UCHL1	Ubiquitination
	PPIF	Protein folding
	IF2A	Protein synthesis
	ATG2A	Autophagosome assembly
	RAB1A	Vesicular trafficking
Cytoskeleton	NFH, NFL, NFM, SPTN2, ACTZ/ ACTR1A, KINH, TBA1A, TBA1C, TBA4A, TBA8, TBB2A, TBB2B, TBAL3, TBB3, TBB4A, TBB4B, TBB5, TBB6, DCTN1, DCTN2	Neurofilaments and Microtubules
Accumulation of abnormal aggregates	APP	Release of Aβ peptides after cleavage
	TDP43	RNA metabolism, accumulation in ALS

We combined 56 proteins common to Aβ-specific aggregates from AD and CVD hippocampi, 7 proteins common to BRI-Aβ and post-MI mouse hippocampi relative to their respective controls (converted to their human counterparts), and 25 proteins that were differential in amyloid AD/AMC and CVD/AMC interactome degree comparisons. The final list, comprising 76 differential proteins after duplicate removal, is shown in the table with their functions. Of these 76, 19 are involved in mitochondrial functions, 2 have ER-related functions, 8 are involved in calcium signaling, 7 have synaptic functions, 5 have apoptotic roles, 14 are involved in proteostasis pathways such as UPS, protein folding, and autophagy, 20 are cytoskeletal components, and 2 are directly involved in abnormal aggregate accumulation (TDP43 and APP).

**Fig. 4:**
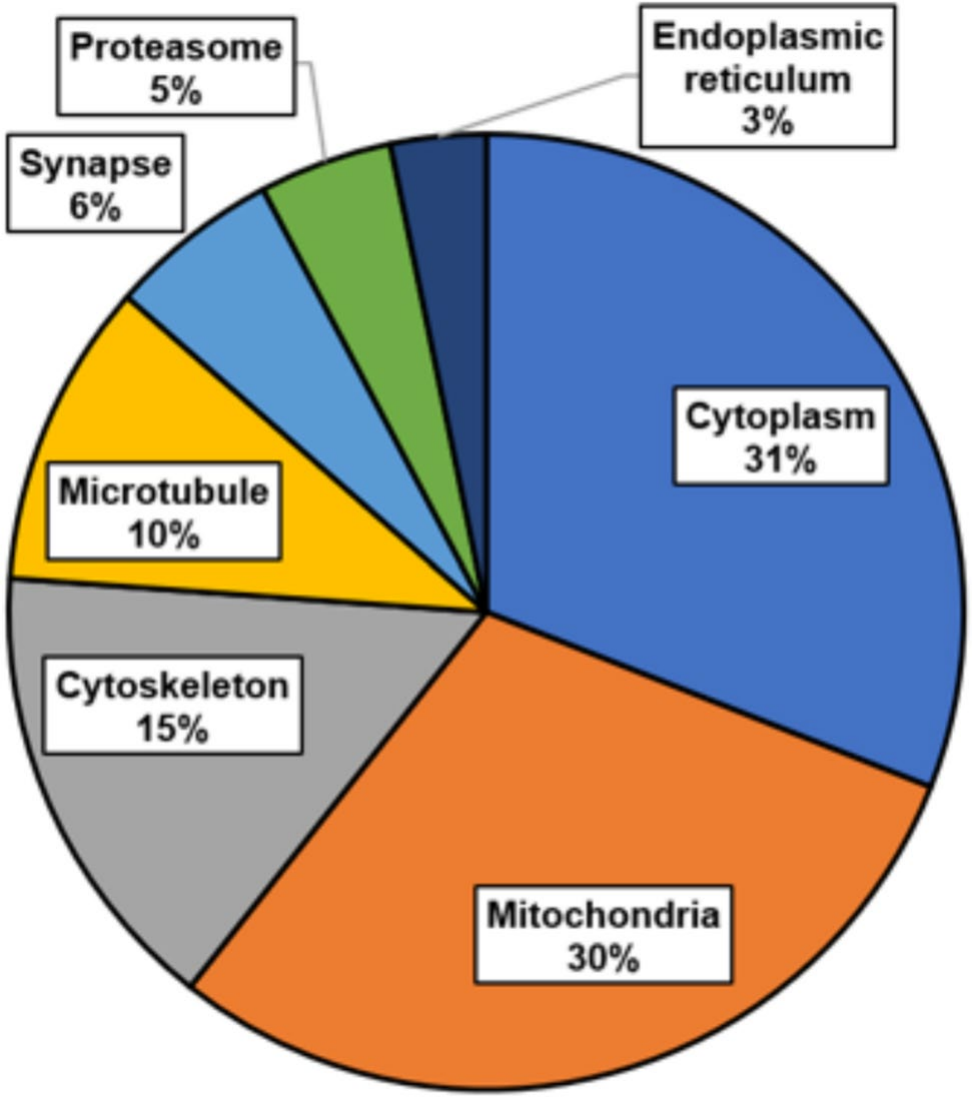
Cellular localization of differential proteins common to CVD and AD aggregates in human hippocampi and mouse models of AD or CVD, based on KEGG pathway analysis. Cellular localizations of 76 differential proteins implicate chiefly cytoplasm and mitochondria, followed by cytoskeleton, microtubule, synapse, proteasome, and sarcoplasmic reticulum

We also determined the cellular localizations of differential proteins using the UP_KW cellular-component subset, implemented within DAVID [18]. We noted that ~ 60% of these proteins localize to cytoplasm and/or mitochondria, while the remainder are associated with cytoskeleton, microtubules, synapses, proteasomes, or endoplasmic reticulum (Fig. 4).

### RNAi knockdowns of differential proteins reduce hypoxia-induced protein aggregation in human cells overexpressing a familial-AD double mutant of APP

We conducted siRNA knockdowns targeting 7 of the 76 differential proteins shared by AD and CVD Aβ-amyloid aggregates. First, we treated human SY5Y-APP_Sw_ cells with siRNAs and then simulated effects of myocardial infarction in vitro by exposing cells to hypoxia. The cells were placed in a hypoxia chamber (5% O_2_, 5% CO_2_ and 90% N_2_) for 7 h and then transferred to a normoxic incubator (21% O_2_, 5% CO_2_) for 16 h to simulate “reperfusion”. This sequence mimics ischemia–reperfusion (I/R) due to myocardial infarction [59, 109]. The cells were harvested 48 h after siRNA treatment and the effect of siRNA KD on aggregation was assessed by thioflavin-T-induced fluorescence.

**Fig. 5:**
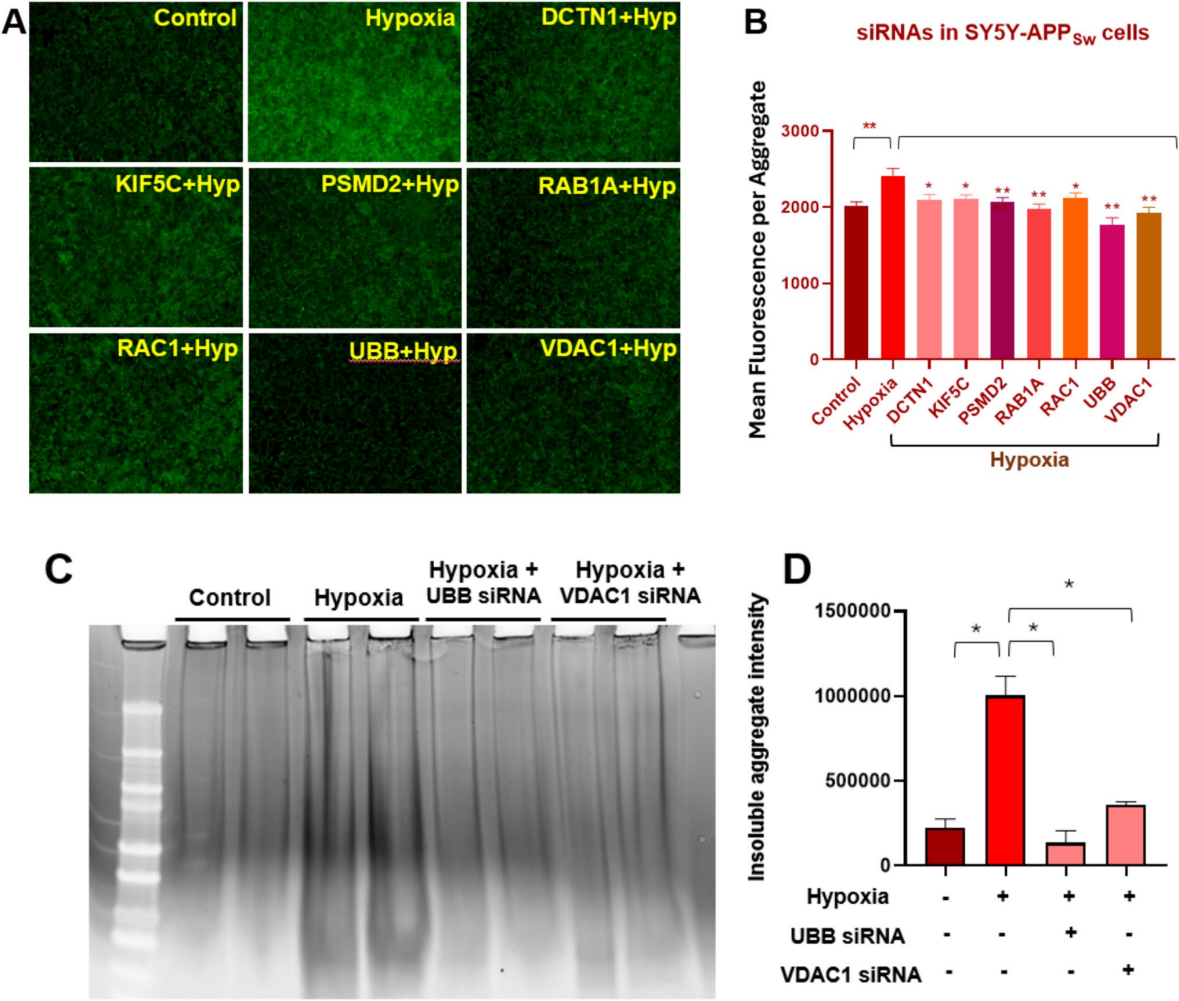
Hypoxia-induced protein aggregation in neuroblastoma cells is reduced by RNAi knockdown of genes encoding highly differential aggregate proteins. After 7 h of exposure to hypoxia, SY5Y-APP_Sw_ cells were transfected with siRNA or shRNA constructs (via RNAiMax lipofection) targeting *DCTN1, KIF5C, PSMD2, RAB1A, RAC1, UBB*, and *VDAC1*. Cells were returned to a normoxic incubator (reperfusion for 48 h), after which they underwent Thioflavin T staining or were harvested for aggregate isolation. **A**, **B** Thioflavin**-**T fluorescence is shown, for SY5Y-APP_Sw_ cells after transfection with siRNAs targeting 7 differential proteins. All knockdowns reduced aggregation significantly. *P < 0.05; **P < 0.01 by 2-tailed heteroscedastic *t* tests. **C**, **D** Detergent-insoluble aggregates from control and anoxia-treated SY5Y-APP_Sw_ cells are reduced tenfold by prior RNAi knockdown with *UBB* siRNA, and 3.5-fold after KD with *VDAC1* siRNA. Reduction in aggregate protein after each siRNA was significant at *P < 0.01 by 2-tailed heteroscedastic *t* tests

We estimate that amyloid content was increased ~ 20% by anoxia (*P* < 0.01) but was restored to roughly control levels by siRNAs targeting *DCTN1*, *KIF5C*, *PSMD2*, *RAB1A*, or *RAC1* (each *P* < 0.05; Fig. 5B). Aggregation was also reduced to levels well below control cells by siRNAs targeting *UBB* and *VDAC1* (each *P* < 0.01; Fig. 5D). These seven target proteins are involved in microtubule architecture, synapse formation, UPS, membrane trafficking, GTPase activity, mitochondrial function, apoptosis, and calcium signaling—functions often disrupted in neurodegenerative diseases. The drugs targeting these proteins may prevent their accumulation in aggregates and thereby avoid or delay the onset of neurodegenerative disease.

### Leave-one-vertex-out analysis of AD and CVD aggregate interactomes, relative to AMC, predicts differential proteins important for aggregation

**Fig. 6:**
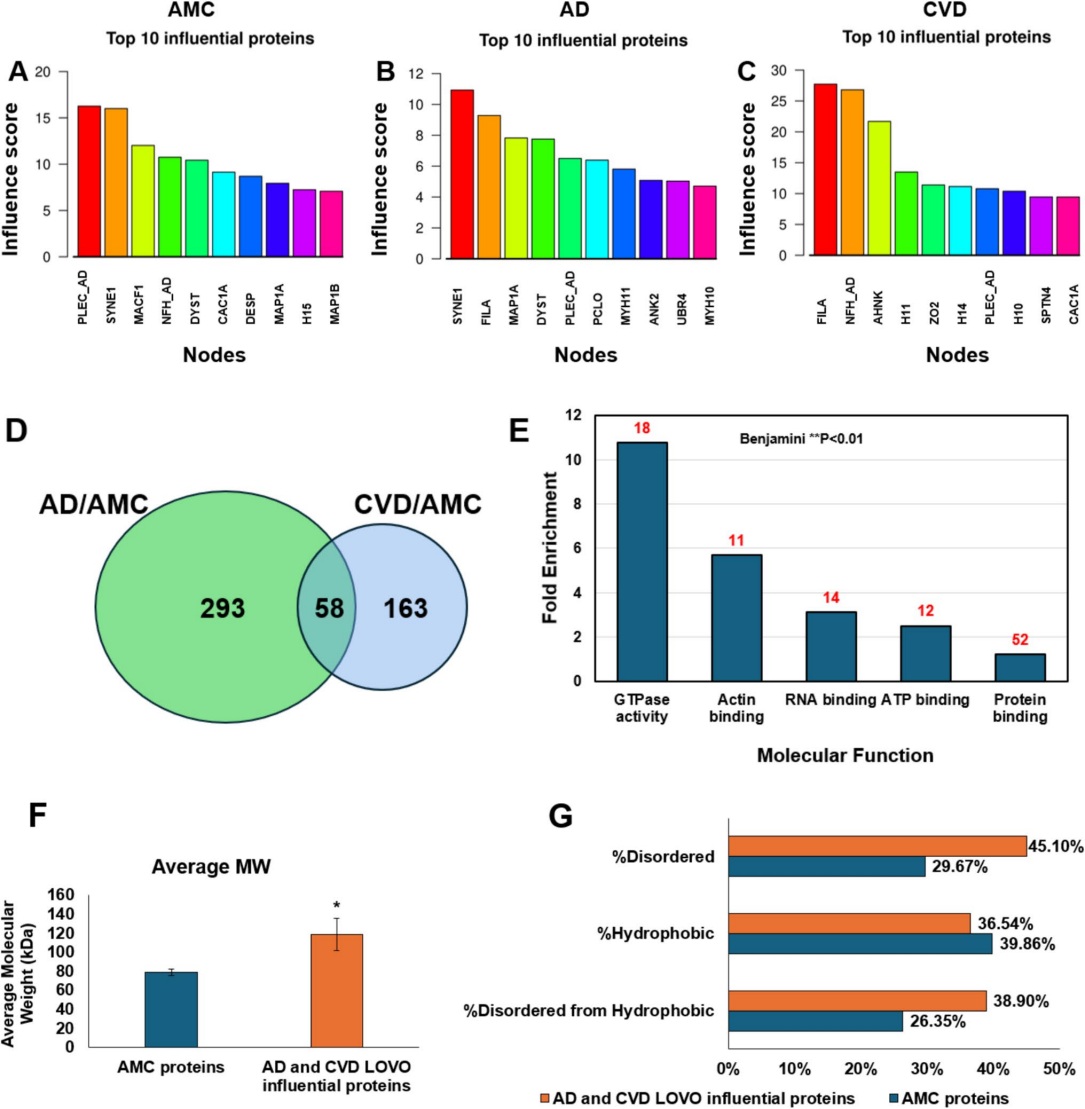
Leave-one-vertex-out analysis (LOVO) in AMC, AD, and CVD interactomes ranks proteins by predicted influence on aggregate stability and growth. Crosslink-based interactomes, constructed for AMC, AD and CVD aggregates, were used to conduct leave-one-vertex-out (LOVO) analysis using R programming. Top influential proteins after LOVO analysis were plotted, and molecular functions and structural properties of influential AD and CVD proteins (each relative to AMC) were listed. **A–C** LOVO analysis of AMC, AD, and CVD interactomes, showing top influential proteins and their influence scores in each interactome. **D** Influential proteins based on higher influence ratio of AD/AMC and CVD/AMC shows 58 common proteins between them. **E** GO term analysis of these common influential proteins implicates functions related to GTPase binding, RNA binding, actin binding, ATP binding, and protein binding with Benjamini-adjusted *P* value < 0.01 for annotation enrichment in DAVID (https://david.ncifcrf.gov/home.jsp). **F** Average molecular weight is increased > 60% in AD and CVD aggregates, relative to AMC (*P < 0.05, by 2-tailed heteroscedastic *t* tests). **G** Percent disordered proteins is elevated > 50% among influential proteins involved in aggregates from AD and CVD consensus interactomes relative to AMC

We used leave-one-out-analysis, implemented with a freely available web-based tool we created (see *simlab.uams.edu/LOOA*) [63]. This program predicts differential proteins and their interactions in aggregate-interactome data by successive removal (with replacement) of one protein or one protein–protein interaction at a time, then determining its influence based on graph modeling calculations of total-aggregate degree [63]. Leave-one-vertex-out (LOVO) analysis predicted the most influential protein nodes in AMC, AD, and CVD interactomes. Many of the top-ranked proteins had been previously implicated in neurodegeneration; for the AD interactome, these include FILA, PLEC, SYNE1, UBR4, and ANK2; while in the CVD interactome we find NFH_AD, FILA, PLEC, CAC1A, and SPTN4 (Fig. 6A‒C).

To clarify the biological implications of LOOA results, we compared AD and CVD influential proteins (based on LOVO analysis) with those from AMC. LOOA influence ratios were derived by dividing AD or CVD influence scores by AMC scores, yielding 351 proteins with positive AD/AMC influence ratios and 218 proteins with positive CVD/AMC ratios. Of these proteins, 58 were common to both comparisons (Fig. 6D). The molecular functions of top proteins are shown in Fig. 6E; they include GTPase activity, actin binding, RNA binding, ATP binding and protein binding, all with significant fold change (each Benjamini *P* < 0.01).

We then determined the average molecular weight, percent hydrophobic residues, and percent disordered residues (using efficient disorder prediction with ESpritz [104]) for 58 shared influential proteins, and normalized each to the corresponding mean for the same protein in AMC. We observed that mean protein size (molecular weight) is ~ 1.5-fold higher in AD and CVD shared influential proteins than in AMC (based on LOVO degree ratios), implying that larger proteins accumulate preferentially in aggregates of AD and CVD brains (Fig. 6F). We also observe that 45% of residues of proteins in the shared influential-protein list are disordered, a 1.5-fold boost over 30% observed for AMC proteins. In contrast, the fractions of hydrophobic residues are similar for influential proteins shared by AD and CVD aggregates, to those in AMC. Of all hydrophobic residues in proteins with positive influence ratios in AD/AMC and CVD/AMC comparisons, 39% are disordered, which is nearly 1.5-fold higher than the disordered fraction (< 27%) in AMC (Fig. 6G).

## Discussion

### Cardiovascular diseases as risk factors for Alzheimer’s disease

Mortality rates due to cardiovascular disease (CVD) have risen significantly over the past decade, surpassing 19.9 million CVD deaths in 2021 [65]. The worldwide incidence of dementia (60–70% of which comprises Alzheimer’s disease) increased from ~ 30 million in 2010 to ~ 55 million in 2020 (www.who.int) and is projected to rise to ~ 150 million dementia diagnoses by 2050, chiefly due to aging of the population [78, 88]. Although AD is more prevalent in aged individuals, it is not an inevitable consequence of aging, even among those genetically predisposed [41, 77, 85]. AD not only creates an immense burden on those afflicted and their families/caregivers, but also affects overall health and negatively impacts the efficacy of medical care. Since no medications have been shown to reverse the pathological changes associated with the disease, most effort has focused on identification of risk factors and biomarkers for early diagnosis of AD. Patients with mild-to-moderate AD symptoms can maintain a reasonable quality of life for years; early diagnosis offers the opportunity to formulate treatment plans and employ therapeutics to slow progression [7, 28, 48]. Established risk factors for AD include the APOE4 allele [19, 55]; genetic variants of amyloid precursor protein (APP), presenilin 1 (PSEN1), and presenilin 2 (PSEN2) [54]; metabolic diseases or conditions such as diabetes and obesity [30, 50]; and vascular conditions such as cardiovascular disease, stroke, and hypertension [15, 21, 32].

Cardiovascular disease (CVD) has been shown in multiple studies to be a prominent risk factor for subsequent dementia [10, 24, 47, 107]. Hypertension, the chief risk factor for CVD, accounts for 2–10 percent of midlife dementia cases worldwide and a study in the UK showed that the incidence of dementia in hypertensive patients has risen from 1.98 to 5.29 per 1000 person-years at risk [1, 71]. Moreover, based on 35 years of follow-up for 314,911 myocardial infarction (MI) patients and 1,573,193 non-MI age-matched controls, ~ 9% of MI patients who suffered stroke or severe heart failure within a year of MI, were eventually diagnosed with dementia or cognitive decline [97]. Meta-analysis of multiple studies implied that coronary heart disease was associated with an increased risk of dementia, with relative risk (RR) of developing dementia ranging from 1.27 to 1.45. [17, 60, 95, 108], while atrial fibrillation raises the RR to 1.44 [20]. We recently reported that MI causes protein misfolding and aggregation in both heart and brain, attributed to ER stress as evidenced by increased levels of GRP78, ATF6, and phospho-PERK in both hearts and brains of induced-MI mice [64]. Phospho-PERK becomes activated via both the ER and mitochondrial unfolded protein responses (UPR^ER^ and UPR^Mit^, respectively). Calcium sequestered in ER is channeled to mitochondria where it normally supports ATP production [53, 76]. Dysfunctional or stressed ER and mitochondria can impair autophagic pathways, calcium signaling, and ATP production [9, 68]. Two protein components of the ubiquitin-proteasomal system (UPS)—ubiquitin-C-terminal hydrolase (UCHL1), a deubiquitinase, and polyubiquitin-B (UBB) —accumulate in post-MI aggregates, contributing to dysfunctional synaptic activity and proteinopathy that arises after cardiovascular disease [57]. Sequestration of critical proteins involved in protein homeostasis, following cardiovascular disease, further augments the burden of misfolded proteins that accumulate in heart and brain aggregates.

Cerebral hypoperfusion and consequent hypoxia, arising after heart failure, worsen both vascular homeostasis and proteostasis of the brain. Chronic hypoxia was shown to increase cellular calcium uptake, mitochondrial calcium content, and disruption of protein folding, all of which contribute to aggregation [52]. Among biomarkers identified for CVD, several are also highly expressed in AD, such as high-sensitivity C-reactive protein (hs-CRP) [51, 62, 100] and galectin-3 (involved in microglial activation) [29]. Although pathways shared by brain aggregation in both CVD and AD have not been extensively studied, cerebral hypoxia is a very common sequela of CVD, known to drive mitochondrial stress, apoptosis, and generation of Aβ_1–42_ peptide, which accompany and may promote protein aggregation [94, 96].

### Systemic fate of amyloid beta and implications in CVD

Amyloid beta (Aβ) is produced from amyloid precursor protein (APP) through enzymatic cleavage, forming soluble oligomers that aggregate into neurotoxic plaques [12]. These plaques contribute to Alzheimer’s disease (AD) by inducing neuroinflammation, oxidative stress, tauopathy, and mitochondrial dysfunction [12, 46]. Aβ is primarily found in the brain but is also produced in peripheral organs including the liver, kidneys, and heart [110]. The brain predominantly contains Aβ_42_, the more aggregation-prone form, while Aβ_40_ is more common in the periphery [110].

Aβ clearance is crucial to determining AD severity. The brain limits Aβ levels through restricted transport across the blood–brain barrier, microglial uptake, and enzymatic degradation [99], while peripheral organs (e.g., liver and kidneys) aid in its clearance from circulation [110]. Systemic immunity, including phagocytes and macrophages, plays a key role, but its function declines with age and AD progression [22]. Diverse systemic conditions—including metabolic disorders, cardiovascular disease (CVD), liver/kidney dysfunction, and chronic inflammation—impair Aβ clearance, increasing AD risk [16, 81, 84].

Aβ_1-40_ is particularly implicated in CVD, promoting vascular inflammation, cardiovascular aging, and atherothrombosis. It is associated with atherosclerosis, acute coronary syndrome (ACS), and heart failure [92]. Conversely, CVD can hinder Aβ clearance by increasing oxidative stress and inflammation, which disrupt cell functions responsible for Aβ clearance, resulting in its buildup in the brain [34, 101]. Targeting Aβ metabolism and its inflammatory effects may offer new therapeutic strategies to protect both the brain and heart.

### Proteinopathies in AD and CVD aggregates

Several previous research studies have identified proteotoxicity in heart and brain of CVD and AD human patients and in their animal models [70, 86, 102, 103]. The plaque-like deposits have been observed in both hearts and brains of individuals with idiopathic dilated cardiomyopathy (iDCM) [31, 102], calcium homeostasis disruption [80], and ER stress increases in failing hearts [64, 105]. In addition, enhancing UPR^ER^ and UPR^mt^ [112], targeting UPS [91], and enhancing autophagy [37, 75] all have the potential to remediate neurodegenerative and cardiovascular diseases. Although expression of specific ER stress markers (ATF6, GRP78, PERK and IRE1), regulators of UPR (heat shock proteins), some UPS proteins, and autophagy-related proteins have been previously studied in the context of CVD and/or AD [11, 114], no direct link has yet been established between AD and CVD. Through the present study, we have identified specific proteins common to AD and CVD aggregates, involved in key proteinopathy pathways (Table 1)—implying that therapeutics targeting the aggregation process may serve as preventive and/or therapeutic measures for multiple age-progressive diseases.

The identification of mitochondrial, ER, calcium-signaling, synaptic, apoptotic, proteostatic, and cytoskeletal proteins accumulating in Alzheimer’s disease and cardiovascular disease aggregates suggests their potential as biomarkers for diagnosis and progression tracking of such diseases.

Mitochondrial dysfunction appears central to both AD and CVD, with NDUA2 and COX2 enriched in aggregates, indicating impaired oxidative phosphorylation and ATP production [83]. The marked enrichment of mitochondrial proteins in pathological aggregates suggests disruption of mitochondrial import and energy deficits, making them potential biomarkers for neurodegeneration.

ER dysfunction is implicated by aggregate enrichment of 14-3-3 paralogs, calcium-dependent protein phosphatases, ELK1, and WNK4 Na^+^/K^+^ transporters, which regulate autophagy and calcium homeostasis [35, 67, 111]. Dysregulated calcium transport and release from the ER further suggest disrupted cellular signaling and excitotoxicity [115], relevant to biomarker development for neurodegenerative diseases.

Markers of synaptic dysfunction in AD and CVD aggregates include glutamate receptor proteins, synaptic transmission regulators, and synaptic vesicle trafficking proteins, linking aggregation to impaired neurotransmission and cognitive decline [73]. Apoptotic proteins, including caspase regulators and kinases that phosphorylate amyloid precursor protein (APP), highlight apoptosis-related neuronal loss as a shared disease mechanism.

Proteostasis markers, such as proteasomal subunits, ubiquitinylation components, and chaperones, accumulate in aggregates, suggesting impaired protein degradation pathways that could be tracked in biofluids [23]. The presence of neurofilament and cytoskeletal proteins in aggregates underscores their role in neuronal-structure instability and axonal degeneration, with neurofilament light chain (NfL) proposed as a promising serum biomarker [14].

**Fig. 7:**
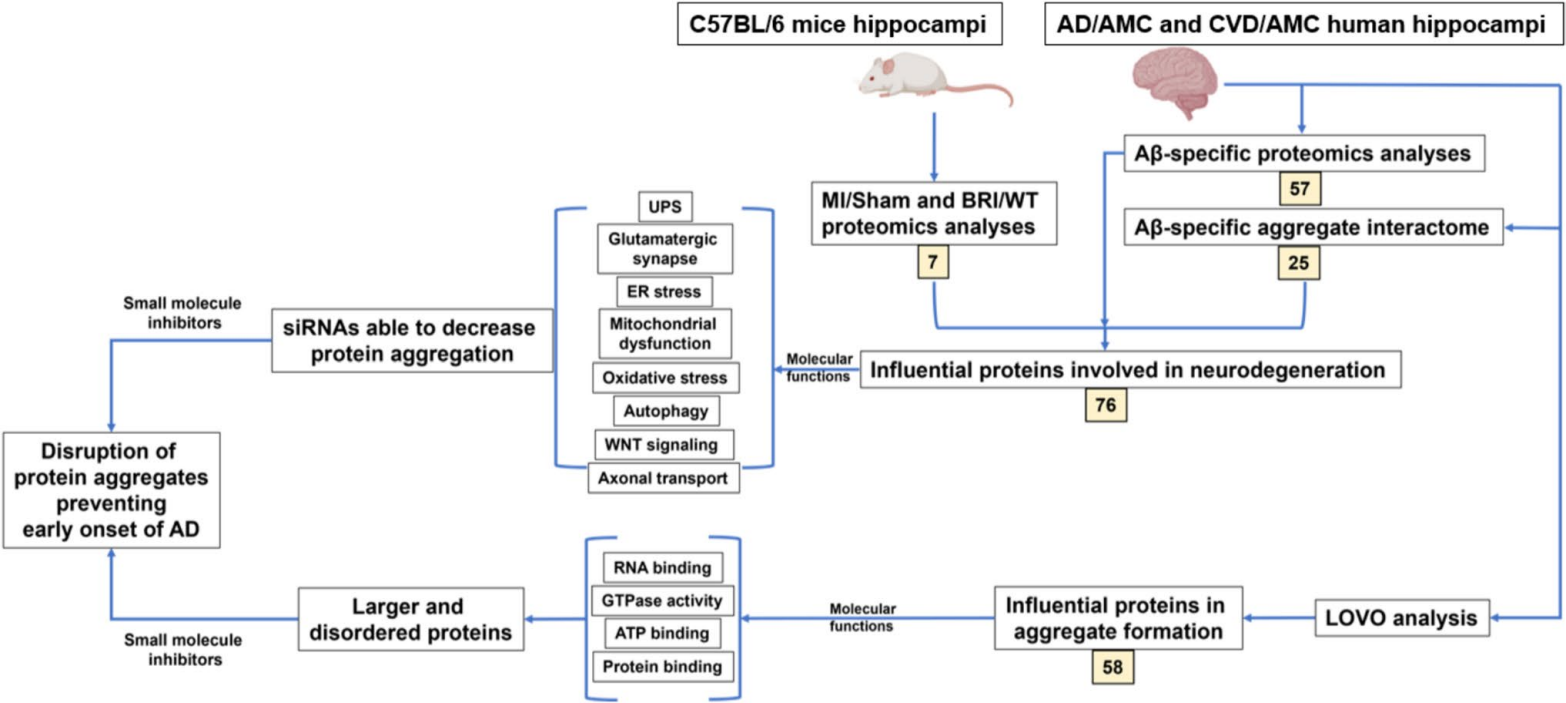
Summary of research implicating differential aggregate proteins in disease models

Overall, the shared aggregation profile between AD and CVD suggests common pathological pathways, highlighting these proteins as potential biomarkers for early detection, disease monitoring, and therapeutic targeting. Future research could leverage these insights to develop biomarker-based diagnostics and neuroprotective treatments for CVD-related neurodegeneration (Fig. 7).

### Aggregate accrual in AD and CVD aggregates

The accrual of protein aggregates in AD has historically been focused chiefly on amyloid beta and tau oligomerization; however, recent evidence suggests that other proteins may initiate or exacerbate aggregation [27, 43, 89, 90]. Our leave-one-vertex-out (LOVO) analysis provides insight into proteins influencing aggregate stability and growth.

LOVO identified 58 key proteins enriched in AD and cardiovascular disease (CVD) aggregates compared to age-matched controls (AMC). These proteins are functionally associated with GTPase activity, actin binding, ATP binding, RNA binding, and protein interactions, and tend to be ~ 1.5 times larger on average than those in AMC aggregates. This size bias suggests that longer translation and folding times, by increasing exposure of hydrophobic cores, may predispose them to misfolding and aggregation [13, 33]. Furthermore, errors in transcription and translation, including nonsense truncations and missense mutations, may increase hydrophobicity and burden proteostasis machinery [93], as evidenced by accumulation of 26S and 20S proteasome subunits, ubiquitination complexes, chaperones, and autophagy-related proteins in AD and CVD aggregates.

Additionally, proteins highly enriched in AD and CVD aggregates exhibited 45% disordered residues, markedly and significantly higher than the 30% level observed in AMC aggregates. Disordered proteins are prone to misfolding, exposing hydrophobic regions that promote inter-protein adhesion and further aggregate growth [3, 26].

LOVO’s ability to identify aggregation-prone proteins presents therapeutic opportunities, particularly in targeting aggregate-promoting proteins. Interventions aimed at stabilizing disordered proteins, or enhancing their degradation through proteostasis mechanisms, could reduce aggregate burden. By elucidating proteins that drive aggregate stability and accrual, LOVO provides a foundation for biomarker discovery and therapeutic targeting to slow neurodegenerative progression in AD and subsequent to CVD.

Altogether, 76 proteins were identified through proteomic and interactome analyses of aggregates from human hippocampi and from mouse models of AD and CVD *vs*. controls. LOVO analysis predicted 58 proteins to be influential for aggregate accrual in AD and CVD vs. AMC. These protein sets were assessed for annotation term enrichment, which implied involvement in cellular functions previously implicated in neurodegeneration. The novel drug targets to oppose aggregate growth were validated by siRNA knockdowns in cultured human cells. Targets most severely impacting aggregation can be used to screen small-molecule structures to discover novel drugs that oppose aggregation.

## Future directions

Future research should focus on validation of shared AD and CVD aggregation-prone proteins as biomarkers for early diagnosis and disease progression in both diseases. Integrating these proteins into multi-modal biomarker panels is expected to enhance diagnostic sensitivity and specificity. Moreover, investigating their roles in aggregate accrual and targeting aggregate-promoting proteins through their stabilization or enhanced degradation may offer novel therapies. The use of computational tools such as LOVO analysis and its further improvement can refine our understanding of aggregate dynamics, paving the way for precision medicine approaches in neurodegenerative disease treatment.

## Limitations

Translation of findings from mouse models to human neurodegenerative-disease pathology is limited by differences in brain structure and complexity, genetics, disease progression, and immune responses, as well as species-specific features of neuroinflammation and proteostasis. Cognitive and behavioral differences make modeling human symptoms difficult, and disparities in drug metabolism may alter therapeutic properties. While mouse models are valuable for studying disease mechanisms, human-based models and clinical trials are essential for effective translation to human pathology.

In addition, comparison of MI vs sham-MI in mice was conducted 1 week after LCA ligation surgery, which is a short time frame to observe significant effects on the brain. Future studies will focus on longer post-ligation intervals to better understand how post-MI aggregation leads to brain proteinopathy. The use of SY5Y-APP_Sw_ cells to study aggregation through RNA knockdown could be expanded by incorporating other cell lines to more accurately represent human-brain cell types. Additionally, isolation of amyloid aggregates with Aβ antibodies could be extended by immune-pulldown of other AD-associated proteins (e.g., tau or hP-tau).

## Supplementary Information

Below is the link to the electronic supplementary material.

Supplementary file1 (DOCX 1043 KB)

## Data Availability

The data will be provided upon written request.
