# Supplementary material for: Altered protein homeostasis in cardiovascular diseases contributes to Alzheimer’s-like neuropathology
Source: Basic Res Cardiol. 2025 May 7;120(3):489–507. doi: 10.1007/s00395-025-01109-w (PMC12158837; doi:10.1007/s00395-025-01109-w)
Supplement: Supplementary file 1 — Supplementary file1 (DOCX 1043 KB) [file 395_2025_1109_MOESM1_ESM.docx]

| **Group** | **ID** | **Age** | **Sex** | **APOE genotype** | **Description/AD severity (1-6)** |
| --- | --- | --- | --- | --- | --- |
| **AMC** | AMC1 | 69 years | Male | 3,3 | NA |
|  | AMC2 | 83 years | Male | 3,3 | NA |
|  | AMC3 | 80 years | Male | 3,3 | NA |
|  | AMC4 | 84 years | Male | 3,3 | NA |
| **AD** | AD1 | 82 years | Male | 4,4 | 5 |
|  | AD2 | 86 years | Male | 4,4 | 4 |
|  | AD3 | 72 years | Female | 4,4 | ND |
|  | AD4 | 90 years | Female | 4,4 | ND |
| **CVD** | CVD1 | 75 years | Male | 2,3 | Coronary Artery Disease/Heart Failure |
|  | CVD2 | 58 years | Male | 3,4 | Coronary Artery Disease |
|  | CVD3 | 69 years | Male | 3,3 | Atherosclerotic Heart Disease |
|  | CVD4 | 77 years | Female | ND | Coronary Artery Disease/Myocardial Infarction |

*NA – Not Available

*ND – Not Determined

**Supplementary Table 1: Metadata for AMC, AD and CVD human hippocampal tissues**


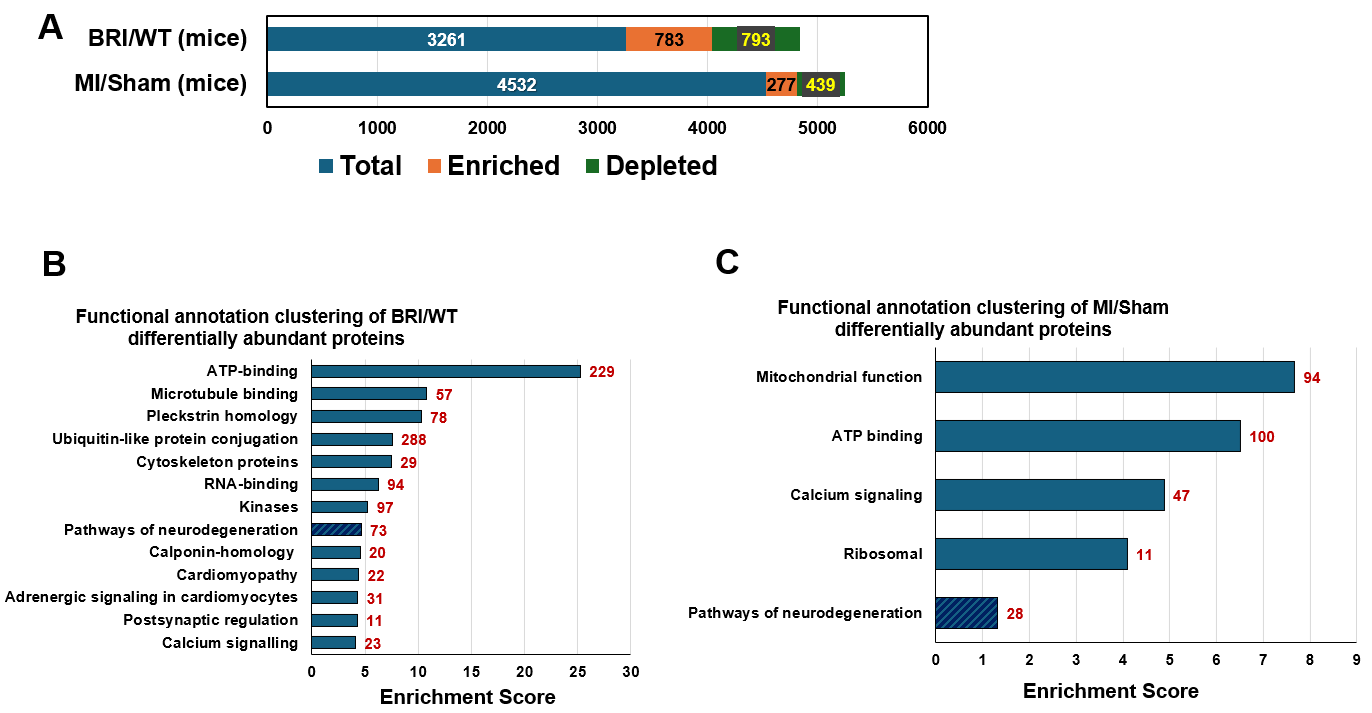


**Supplementary Figure 1**: Enriched and depleted proteins and functional annotation clustering of differentially abundant proteins in mouse hippocampal aggregates. **A**. 783 proteins were enriched, and 793 proteins depleted, in BRI-Aβ_42_ aggregates relative to wildtype (WT). Similarly, 277 proteins were enriched, and 439 proteins depleted, in MI aggregates relative to sham-MI controls.

**B**. Functional annotation clustering using DAVID to determine pathways involving 1576 proteins differentially abundant in BRI-Aβ_42_ *vs.* wildtype mouse hippocampal aggregates. **C**. Functional annotation clustering using DAVID to determine pathways involving 716 proteins differentially abundant in MI/sham-MI mouse hippocampi.


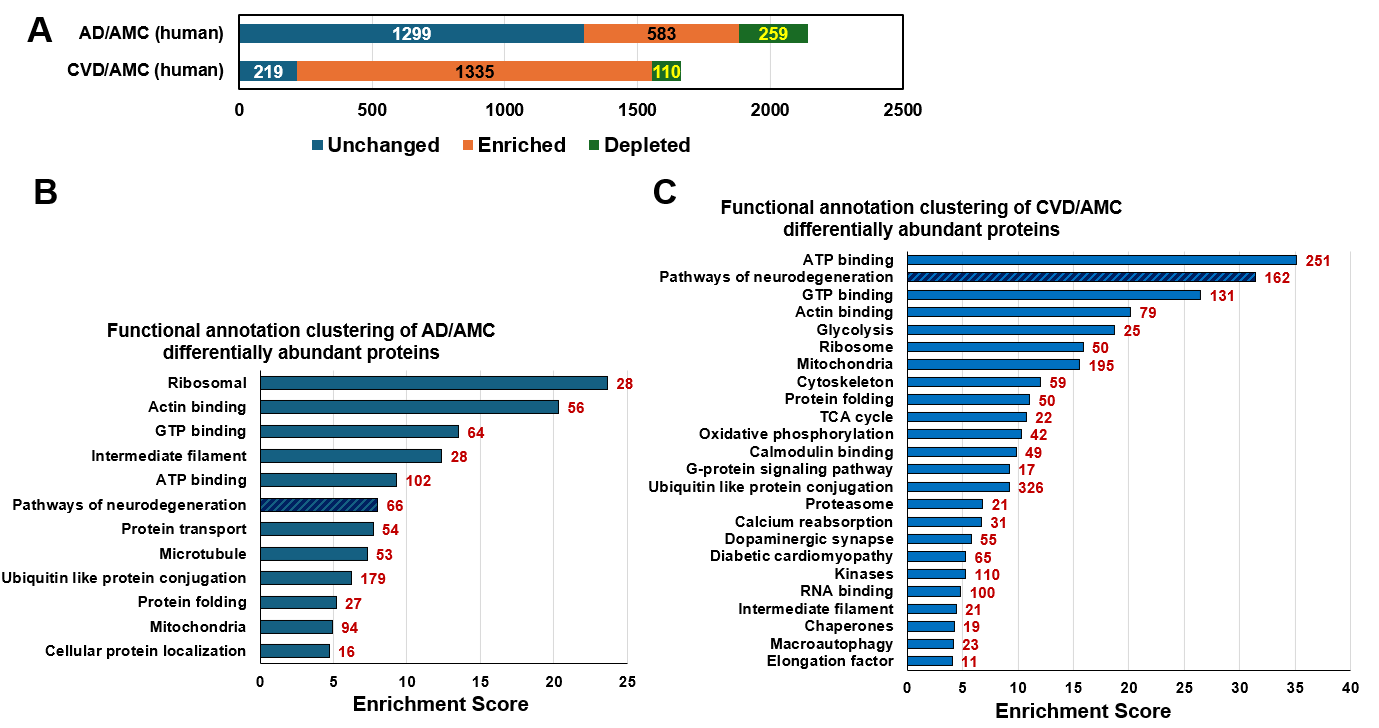


**Supplementary Figure 2**: Enriched and depleted proteins and functional annotation clustering of differentially abundant proteins in human hippocampal aggregates. **A**. We identified 583 proteins enriched, and 259 proteins depleted, in AD relative to AMC aggregates. Similarly, 1335 proteins are enriched, and 110 proteins depleted, in CVD relative to AMC. **B**. Functional annotation clustering using DAVID to determine pathways involving 842 differentially abundant proteins in AD *vs.* AMC hippocampi. **C**. Functional annotation clustering using DAVID to identify pathways involving 1445 hippocampal-aggregate proteins differentially abundant in CVD *vs*. AMC.


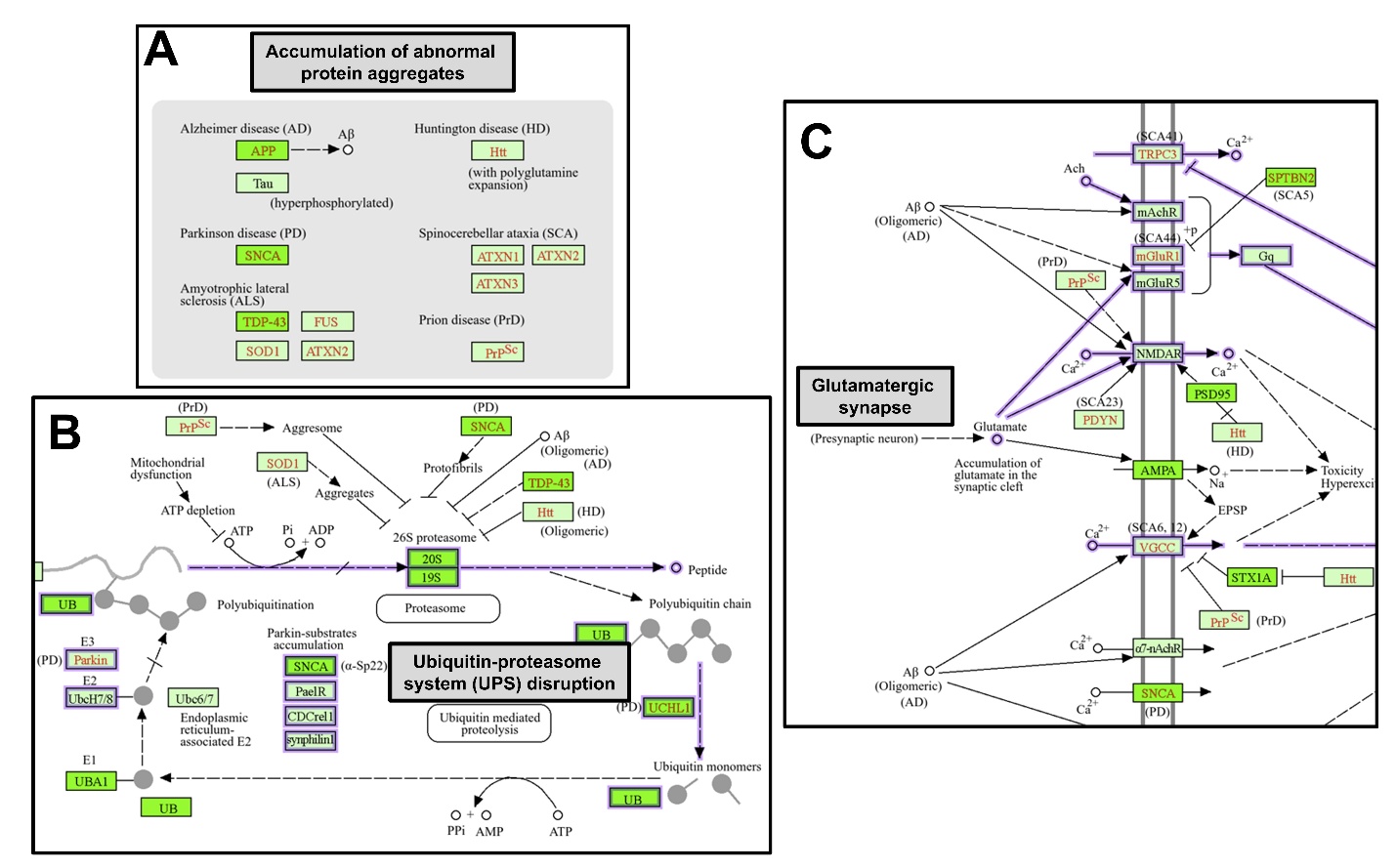


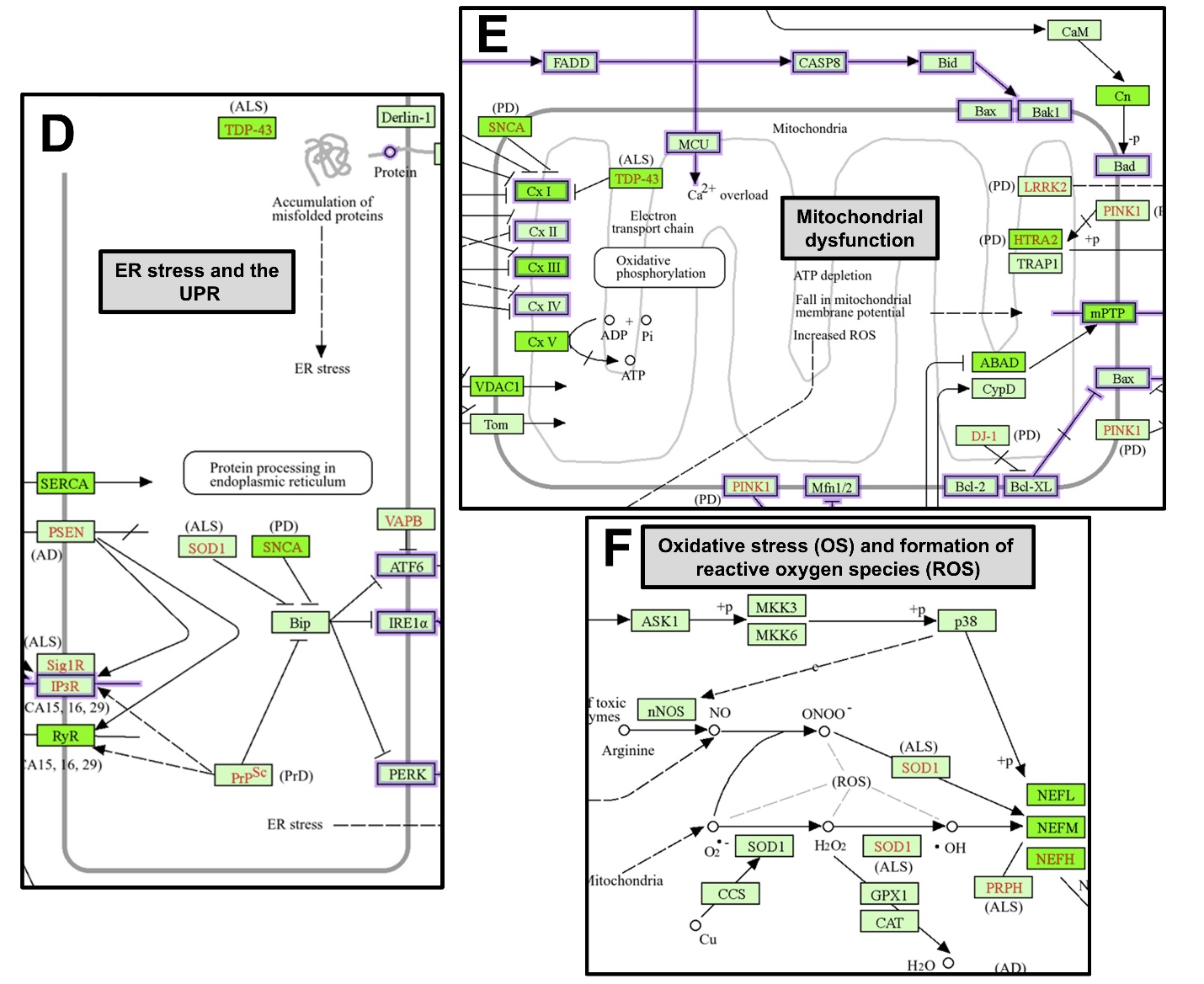


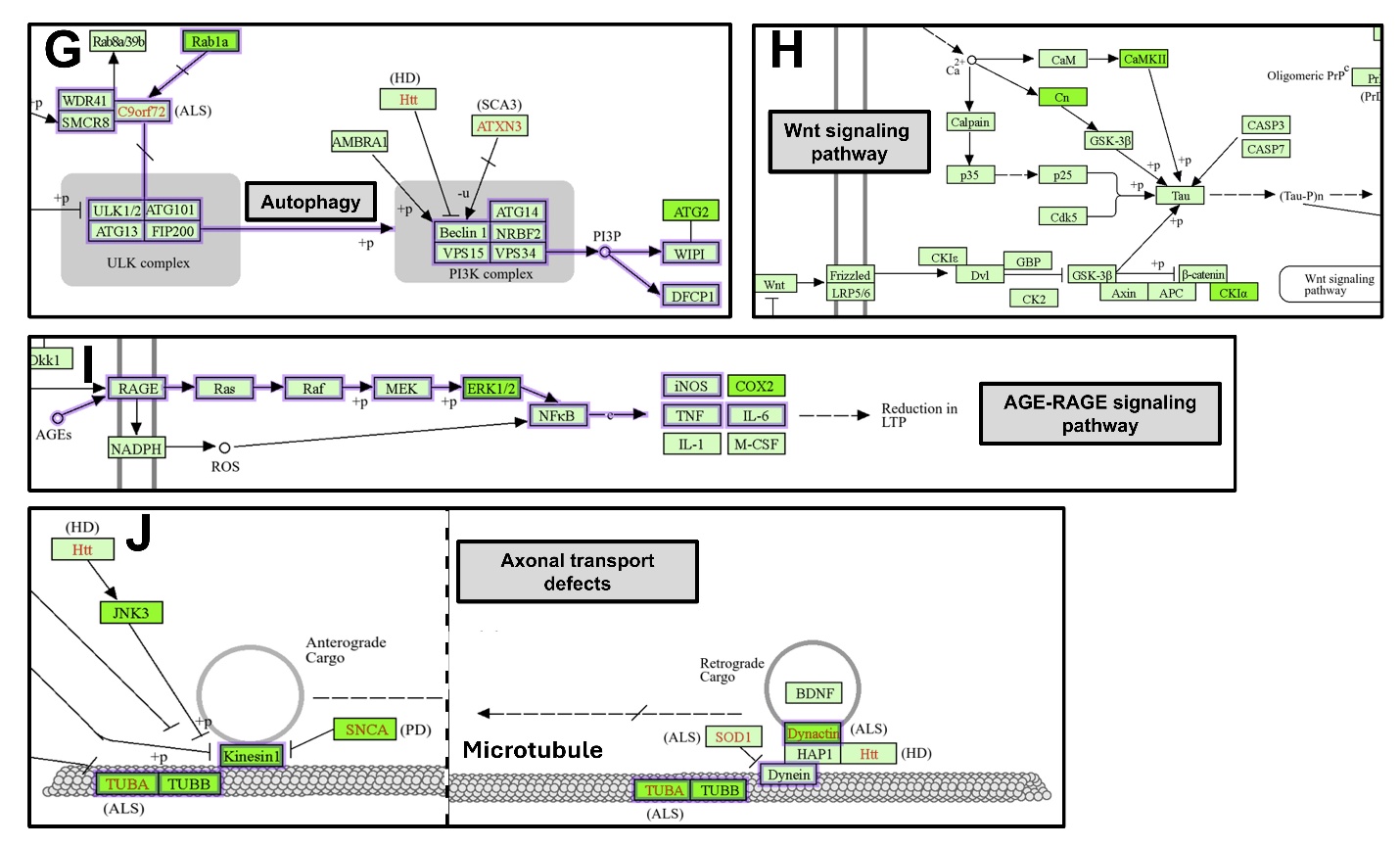


**Supplementary Figure 3**: Neurodegeneration pathways involving 76 influential proteins identified by analyzing AD and CVD mouse models and human hippocampi. **A, B**, **C**: Proteins involved in accumulation of abnormal aggregates, UPC and glutamatergic synapse. **D, E, F**: Proteins involved in ER stress (UPR), mitochondrial dysfunction, and oxidative stress. **G, H, I, J**: Proteins involved in autophagy, Wnt and AGE-RAGE signaling, and axonal transport pathways.
